# Microbiome dysbiosis and therapeutic restoration in atopic dermatitis

**DOI:** 10.3389/fcimb.2026.1693905

**Published:** 2026-02-13

**Authors:** Ling Zhong, Xiyuan Zhou, Jia Su, Yue Zhang, Dingding Zhang, Huiying Wan

**Affiliations:** 1 Department of Dermatology, Sichuan Provincial People’s Hospital, School of Medicine, University of Electronic Science and Technology of China, Chengdu, China; 2 School of Medicine and Life Science, Chengdu University of Traditional Chinese Medicine, Chengdu, China; 3 Sichuan Provincial Key Laboratory for Genetic Disease, Sichuan Provincial People’s Hospital, University of Electronic Science and Technology of China, Chengdu, China

**Keywords:** atopic dermatitis, bacteriotherapy, *Cutaneous Malassezia*, microbiome, *Staphylococcus aureus*

## Abstract

Atopic dermatitis (AD) is increasingly recognized as a chronic inflammatory skin disease driven by a self-reinforcing vicious cycle involving skin barrier dysfunction, immune dysregulation, and cutaneous microbiome dysbiosis. A hallmark of this dysbiosis is the overrepresentation of pathogens like *Staphylococcus aureus* and *Malassezia* species, alongside a marked loss of microbial diversity, particularly during disease flares. This review systematically dissects the host-derived factors—such as altered lipid profiles, reduced antimicrobial peptides, and elevated skin pH—that facilitate *S. aureus* colonization. We further examine how bacterial virulence factors amplify type 2 inflammation and impair barrier integrity, thereby sustaining the pathological loop. We also explore the emerging roles of the skin virome, particularly the phageome, and discuss how microbiome-targeted interventions, including bacteriotherapy with commensal bacteria and precision phage therapy, offer promising avenues for ecological restoration. Finally, we argue that future research must leverage multi-omics to understand strain-specific functions, ultimately guiding the development of personalized microbiome interventions for AD.

## 1 Introduction

Atopic dermatitis (AD), also known as eczema or atopic eczema, is a chronic, relapsing inflammatory skin disease that imposes a considerable socioeconomic and psychological burden worldwide (Kiyomatsu-Oda et al., 2018; Langan et al., 2020). About 80% of cases occur in infancy or childhood, while the rest develop in adulthood (Weins et al., 2024). The 1-year period prevalence in children varies between 4.1% and 22.7% across countries, while in adults, it ranges from 3.5% to 4.9% (Raimondo and Lembo, 2021). Clinically, AD presents with heterogeneous eczematous lesions (e.g., erythema, papules, exudation) in characteristic age-dependent distributions, influenced by skin dryness (Saeki et al., 2025). Although the pathophysiology of AD is not yet fully understood, it is widely recognized to be complex and multifactorial, primarily involving mutations in epidermal differentiation genes, dysfunction of the skin barrier, and immune dysregulation (Sroka-Tomaszewska and Trzeciak, 2021). In addition to local skin factors, accumulating evidence also highlights the role of the gut–skin axis, where gut microbiota dysbiosis may influence systemic immunity and thereby affect AD development (Park DH. et al., 2021). Recently, new insights into the pathophysiology of AD have focused on the relationship between the skin microbiome and the disease (Kim et al., 2019b; Tsakok et al., 2019; Sroka-Tomaszewska and Trzeciak, 2021).

The skin, as the body’s outermost layer, serves as the first line of defense against external pathogens and environmental insults. However, it also hosts a diverse microbiome composed of bacteria, fungi, viruses, and micro-eukaryotes, collectively known as the skin microbiome. Most of these microorganisms are commensals (Flowers and Grice, 2020; Harris-Tryon and Grice, 2022).The skin provides a protective environment for microbial survival, fostering competition and cooperation, and supplying essential nutrients (Flowers and Grice, 2020). The skin microbiome plays a crucial role in protecting the host from harmful microorganisms, a function known as colonization resistance (Flowers and Grice, 2020). This is achieved by directly inhibiting or altering the virulence of pathogens through the secretion of antimicrobial peptides (AMPs) and other molecules (Flowers and Grice, 2020; Rademacher et al., 2021). Additionally, some microbial species stimulate keratinocytes, signaling the host to mount an immune response (Flowers and Grice, 2020). The balance of microbial-microbial and microbial-host interactions is essential for maintaining skin health (Flowers and Grice, 2020; Uberoi et al., 2021). Beyond its role in disease, emerging evidence indicates that the skin–microbiome axis also plays a critical role in maintaining skin homeostasis throughout the lifespan. Age-related shifts in microbial composition can promote local inflammation, oxidative stress, and activation of matrix metalloproteinases (MMPs), thereby accelerating the degradation of extracellular matrix components such as collagen and elastin and contributing to wrinkle formation. These findings underscore the broad significance of microbial regulation in preserving skin health (Challa et al., 2025). However, this complex host–microbiome relationship is a delicate balance; disruptions can lead to dysbiosis and contribute to skin diseases.

Evidence suggests that AD patients’ lesional skin harbors an abundance of *Staphylococcus aureus* (*S. aureus*) and shows reduced microbial diversity (Bjerre et al., 2017; Di Domenico et al., 2019; Bjerre et al., 2021; Fölster-Holst, 2022; Huang et al., 2024). Understanding how microbial communities contribute to AD during the flare cycle is essential. In this review, we examine the development of the skin microbiome and systematically analyze the microbial composition, interspecies interactions, and immunological mechanisms associated with AD. With a focus on elucidating the underlying pathogenesis of the disease and highlighting the potential clinical applications in the development of precision therapeutic strategies.

## 2 Skin microbiome in healthy skin

The skin is home to millions of bacteria, fungi and viruses that make up the skin microbiome (Chen and Tsao, 2013; Karin et al., 2023). With the growing recognition of the pivotal role of the skin microbiome in maintaining cutaneous homeostasis and contributing to disease pathogenesis, the continuous advancement of sequencing technologies has provided essential technical support for research in this field (Byrd et al., 2018). Early first-generation sequencing technologies, such as Sanger sequencing, offer high accuracy but are limited by low throughput and high cost. Consequently, they have been primarily applied to single-gene analyses or microbial identification in a small number of samples, making them inadequate for comprehensive profiling of complex microbial communities (Singer et al., 2016). Subsequently, second-generation sequencing technologies, such as 454 pyrosequencing and high-throughput Illumina sequencing, substantially increased sequencing throughput and depth, enabling systematic elucidation of the ecological distribution patterns of microbiota across different skin sites. Recently, second-generation shotgun metagenomic sequencing has enabled comprehensive, amplification-free profiling of bacteria, fungi, viruses, and other microbes at strain-level resolution, offering new insights into microbiota–skin barrier–immune interactions (Grogan et al., 2019). Meanwhile, third-generation sequencing technologies, such as PacBio and Oxford Nanopore, with their long-read capabilities, offer a complementary approach for high-resolution analysis of complex microbial genome structures and functional heterogeneity (Karin et al., 2023). Overall, the iterative advancement of sequencing technologies has markedly enhanced our understanding of the ecological characteristics of the skin microbiome and its immunological significance. The summary of sequencing technologies commonly used in skin microbiome research and their relevant immunological associations is presented in **Table 1**.

**Table 1:** Comparison of first-, second-, and third-generation sequencing technologies.

Sequencing technologies	Representative technologies	Advantages	Limitations	Applications
First-generation sequencing technologies	Sanger sequencing	High reliability, long read length, and high accuracy	Low throughput, high cost, and slow speed	Determines microbial taxonomy; used for small genomic fragments and key genes; limited for resolving complex microbial evolutionary relationships
Second-generation sequencing technologies	454 pyrosequencing; Illumina next-generation sequencing	High throughput, relatively low cost, and rapid generation of large-scale sequencing data	Short read lengths, typically a few hundred base pairs, limiting resolution of long repeats and complex genomic regions	Provides microbial community composition and relative abundances; generates draft genomes and identifies functional genes; allows multi-gene or whole-genome sequencing for improved evolutionary and phylogenetic analysis
Third-generation sequencing technologies	PacBio; Oxford Nanopore	Long read lengths; PCR-free, allowing direct RNA sequencing and minimizing biases from reverse transcription	Lower throughput than second-generation sequencing, relatively higher error rates (correctable with increased coverage), and higher cost	Enables accurate genome assembly for taxonomic identification; characterizes gene structures and regulation; supports studies of gene expression, specialized functions, structural variation, and long-term microbial genome dynamics

The composition of the skin microbiome varies substantially across different body sites, primarily reflecting the skin’s physiological characteristics. Moist, dry, and sebaceous microenvironments each harbor distinct microbial communities (Costello et al., 2009; Grice et al., 2009; Grice and Segre, 2011; Findley et al., 2013; Oh et al., 2014; Byrd et al., 2018). For instance, *Cutibacterium* species predominate in sebaceous areas, while *Corynebacterium* and *Staphylococcus* species are more abundant in moist regions like the elbow and foot creases (Findley et al., 2013; Lunjani et al., 2019; Requena and Velasco, 2021; Hou et al., 2022). Unlike bacteria, the fungal community shows less variation across different body sites. *Malassezia* species dominate central areas and arms, while the feet exhibit higher fungal diversity and lower stability (Gao et al., 2010). Eukaryotic viruses are transient and show little site-specificity during colonization (Oh et al., 2016).

Evidence suggests that the skin microbiome undergoes dynamic shifts throughout the human lifespan. At birth, delivery mode shapes colonization: vaginally delivered infants acquire vaginal microbes (e.g., *Lactobacillus*, *Prevotella*, *Leptotrichia*), whereas cesarean-delivered infants acquire skin-associated microbes (e.g., *Staphylococcus*, *Corynebacterium*) *(*Dominguez-Bello et al., 2010; Oh et al., 2012; Mueller et al., 2015; Chu et al., 2017; Murphy et al., 2023). Sexual maturity impacts the composition of the skin microbiome (Capone et al., 2023), with sexually immature children having lower relative abundances of *Corynebacterium* and *Cutibacterium*, but greater fungal diversity than adults (Oh et al., 2012; Jo et al., 2016). By late childhood, the skin microbiome gradually stabilizes and reaches a relatively consistent state (Jo et al., 2016). During puberty, the density of lipophilic *Cutibacterium acnes* rises in parallel with sebum production, whereas its abundance is markedly reduced in elderly skin (Challa et al., 2025). It should be noted that *C. acnes* is a heterogeneous species, with some strains providing significant benefits to the skin, while others may promote inflammatory responses (Cros et al., 2023). Meanwhile, potentially pathogenic taxa such as *Corynebacterium* and *Staphylococcus* show a relative increase in elderly skin (Kim HJ. et al., 2019; Li et al., 2020). Additionally, studies have reported a decline in the abundance of Actinomycetes in aged skin (Li et al., 2020). These shifts in microbial composition are influenced by reduced sebum secretion, loss of barrier function, and immunosenescence, collectively leading to microbial dysbiosis and altered host–microbe interactions (Shibagaki et al., 2017; Challa et al., 2025).

Environmental factors and individual lifestyle choices significantly contribute to inter-individual differences in skin microbiome composition and maturation. The residential setting—urban versus rural—significantly influences microbial community structure, particularly in early life (Callewaert et al., 2020). While overall microbial richness is similar between urban and rural populations, rural individuals show greater within-group variability, whereas *Trabulsiella* is more abundant in urban populations (Ying et al., 2015). Research also indicates that cosmetic product use modulates the skin microbiome. A study involving 25 Korean women demonstrated that continuous application of the same skincare product for four weeks led to an increase in facial bacterial diversity and significant alterations in the microbial community structure. The relative abundances of *Cutibacterium* and *Staphylococcus* increased, accompanied by improvements in skin hydration and texture (Hwang et al., 2021). Also, intrinsic factors such as sebum secretion, immune function, and hormonal levels also influence the maturation of the skin microbiome (Challa et al., 2025).

## 3 Skin microbiome in AD

### 3.1 Staphylococcus aureus

**Figure 1:**
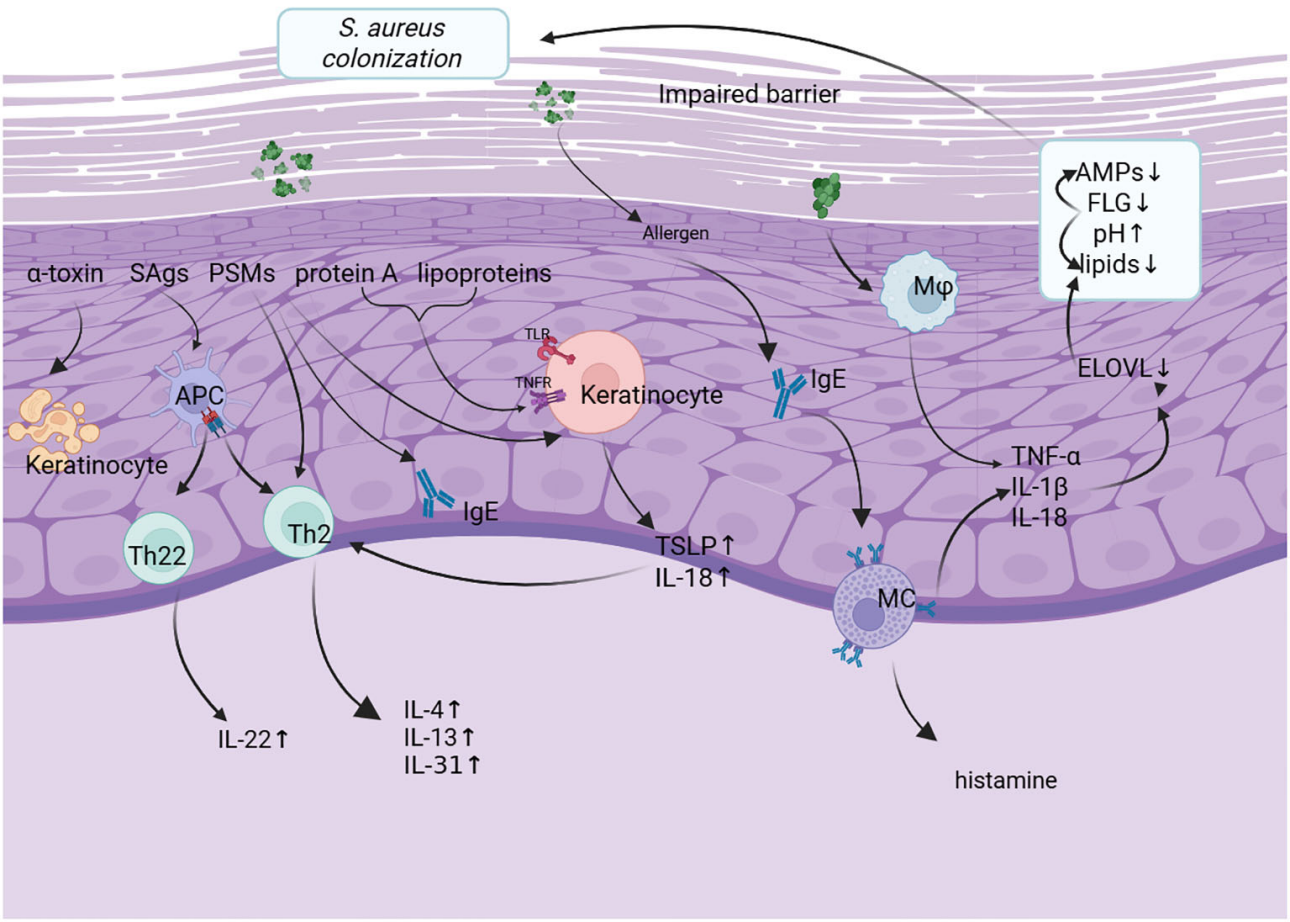
Patients with atopic dermatitis (AD) typically exhibit impaired skin barrier function, characterized by reduced antimicrobial peptides (AMPs), filaggrin (FLG) deficiency, elevated pH, and decreased lipid synthesis. These alterations interact with each other to facilitate the colonization of *Staphylococcus aureus* (*S. aureus*)on the epidermal surface. S. aureus produces multiple virulence factors, including α-toxin, staphylococcal superantigens (SAgs), phenol-soluble modulins (PSMs), protein A(SpA), and lipoproteins, which further disrupt the skin barrier. Among them, SpA and lipoproteins activate Toll-like receptors (TLRs) and tumor necrosis factor receptors (TNFRs) on keratinocytes, leading to the production of proinflammatory cytokines such as thymic stromal lymphopoietin (TSLP) and interleukin-18 (IL-18). SAgs activate antigen-presenting cells (APCs), promoting the polarization of Th2 and Th22 cells and driving the secretion of cytokines such as IL-4, IL-13, IL-31, and IL-22, thereby exacerbating inflammation and barrier impairment. Meanwhile, PSMs can activate specific immune pathways, resulting in increased IgE and IL-4 levels. Additionally, α-toxin exerts cytotoxic effects on keratinocytes through pore formation. Activated macrophages (Mφ) and mast cells (MCs) further contribute to local inflammation by releasing TNF-α, IL-1β, IL-18, and histamine. These mechanisms collectively form a vicious cycle of inflammation, barrier dysfunction, and microbial colonization, driving the progression of AD. *S.aureus*, Staphylococcus aureus; SAgs, staphylococcal superantigens; PSMs, phenol-soluble modulins; APC, Abbreviations antigen presenting cell; Ig E, immunoglobulin E; TLR, toll-like receptor; TNFR, tumor necrosis factor receptor; IL, interleukin; Th, T helper; TSLP, thymic stromal lymphopoietin cell; Mφ, macrophage; ELOVL, elongation of very long chain fatty acids; AMP, antimicrobial peptide; FLG, filaggrin.

*S. aureus* plays a critical role in the development of AD (**Figure 1**). Evidence indicates that bacterial diversity decreases at predilection sites, with an increased abundance of *Staphylococcus* species, particularly *S. aureus* (**Table 2**). The colonization rate of *S. aureus* is approximately 70% in lesional skin and 39% in non-lesional skin (Carmona-Cruz et al., 2022). Although the exact mechanisms remain incompletely understood, one study demonstrated that applying an AD-derived strain of *S. aureus* to wild-type mouse skin induced epidermal hyperplasia, inflammatory responses, and immune cell infiltration—primarily involving neutrophils and eosinophils. This finding highlights the role of *S. aureus* in AD pathogenesis (Williams and Gallo, 2015; Byrd et al., 2017; Gonzalez et al., 2017; Aguilera et al., 2020).

**Table 2:** Comparison of major differences in skin microbiome between healthy skin and AD skin.

Feature	Healthy skin	AD skin
Microbial Diversity	high	significantly reduced
Dominant Bacteria	*Staphylococcus* spp.*, Corynebacterium* spp.*, Cutibacterium* spp.	*Staphylococcus* spp. (particularly S. aureus), *Malassezia* spp.
*S. aureus*	low abundance	significantly increased (approximately 70% in lesional skin)
*Staphylococcus epidermidis*	Commensal bacteria, relatively abundant	decreased relative abundance
Fungal community	low abundance, limited variability, and high stability	*Malassezia* spp. *colonization increases with AD severity*
Metabolic characteristics	anaerobic metabolism	aerobic metabolism
Immune Response	balanced immune response	skewed Th2/Th22 response; increased inflammation
Skin Barrier Function	Intact	Impaired
Antimicrobial Peptide Levels	normal levels	reduced

#### 3.1.1 Host factors promoting colonization

The increased colonization and persistence of *S. aureus* on AD skin are facilitated by a constellation of host-derived factors that impair skin barrier function and weaken innate immune defenses. Several factors promote *S. aureus* colonization in AD, including changes in skin lipids and fatty acids, reduced production of AMPs, mutations in filaggrin (FLG), and a shift in skin pH to more alkaline levels (Ong et al., 2002; Kezic et al., 2011; Brown and McLean, 2012; Chieosilapatham et al., 2017; Niyonsaba et al., 2017; Geoghegan et al., 2018; Clausen et al., 2019; Ogonowska et al., 2020).

The major lipids in the stratum corneum, including ceramides, free fatty acids, and cholesterol with its esters, are essential for maintaining skin barrier integrity (Li et al., 2016). In AD, type 2 inflammatory cytokines such as IL-4, IL-13, and IL-31 are upregulated and have been shown to suppress the expression of key lipid-synthesizing enzymes, thereby further compromising the barrier (Danso et al., 2017; Çetinarslan et al., 2023). Skin fatty acids also serve as important components of the innate immune defense, among which cis-6-hexadecenoic acid (C6H) is considered the most potent fatty acid on the skin surface against *S. aureus* (Takigawa et al., 2005). C6H exerts its antibacterial effect by increasing bacterial membrane fluidity and acting as a proton carrier to disrupt bacterial metabolism, with enhanced activity in acidic environments (approximately pH 5.5) (Cartron et al., 2014). However, its levels are significantly reduced in AD skin, facilitating *S. aureus* overgrowth. Additionally, recent findings suggest that *S. aureus* infection can indirectly inhibit the activity of the Elongation of Very Long Chain Fatty Acids (ELOVL) family enzymes by inducing pro-inflammatory cytokines such as IL-1β and TNF-α, further disrupting the composition of stratum corneum lipids and establishing a vicious cycle of infection, inflammation, and barrier damage (Kim et al., 2023).

AMPs are primarily synthesized by keratinocytes in the skin and exert antimicrobial effects by disrupting the microbial cell membrane or penetrating the membrane to interfere with intracellular processes (Frohm et al., 1997; Harder et al., 1997). However, in the inflammatory skin lesions of patients with AD, the expression levels of AMPs, such as human β-defensin-2 (HBD-2) and LL-37, are significantly reduced (Ong et al., 2002). This reduction is closely associated with the overexpression of type 2 helper T cell (Th2)-associated cytokines, particularly interleukin-4 (IL-4) and interleukin-13 (IL-13), which suppress the transcriptional activity of genes encoding these AMPs (Ong et al., 2002).

AD originates from a genetic predisposition to epidermal barrier dysfunction, such as defects in the FLG gene encoding FLG (Geoghegan et al., 2018). Acute flares are typically triggered when allergens or other irritants penetrate the compromised skin barrier and are processed by antigen-presenting cells in the skin, such as Langerhans cells (Geoghegan et al., 2018). Th2-associated cytokines, including IL-4, IL-13, and IL-25, together with the Th22-associated cytokine IL-22, have been shown to downregulate FLG expression in keratinocytes, thereby playing a critical role in the pathogenesis of AD (Fenner and Silverberg, 2018). Experimental studies using murine AD models have demonstrated that FLG deficiency not only disrupts keratinocyte architecture but also impairs lipid secretion processes (Elias et al., 2017). Consequent reductions in cutaneous lipid levels further diminish the synthesis of AMPs, thereby contributing to alterations in the skin microbiome composition (Langan et al., 2020). Such dysbiosis is exemplified by a markedly increased colonization of pathogenic bacteria, notably *S. aureus* (Clausen et al., 2017). Nevertheless, it is noteworthy that FLG mutations are not universally observed in all AD patients, and a subset of individuals carrying FLG mutations may not present with significant microbial dysbiosis (Nakatsuji et al., 2016; Williams et al., 2020).

The surface of healthy skin is relatively dry and maintains a mildly acidic environment, with a typical pH ranging from 4 to 6 (Geoghegan et al., 2018). The outermost layer of the epidermis, the stratum corneum, undergoes continuous renewal and desquamation, facilitating the removal of adherent microorganisms (Geoghegan et al., 2018). Serine proteases within the stratum corneum, such as the stratum corneum chymotryptic enzyme, exhibit maximal activity at approximately pH 8.0; while their controlled activity supports normal desquamation, elevated pH levels result in excessive protease activation, compromising corneocyte cohesion and stratum corneum integrity, thereby weakening barrier function (Rippke et al., 2004). Moreover, alkaline pH triggers proinflammatory cytokines such as IL-1α and TNF-α, promoting inflammation and further compromising barrier function (Abou Alaiwa et al., 2014). Acidic pH also inhibits the growth of pathogens like *S. aureus* (Rippke et al., 2004). Therefore, increased skin pH disturbs microbial homeostasis, promotes pathogen colonization, and exacerbates inflammation and barrier damage.

#### 3.1.2 Virulence factors and immune activation

Once established on the skin, *S. aureus* stimulates immune responses by expressing various virulence factors. Superantigens (SAgs) produced by all strains of *S. aureus*, including toxic shock syndrome toxin-1 (TSST-1) and staphylococcal enterotoxins (SEA-SEG), play a significant role in AD pathogenesis (Lina et al., 2004; Spaulding et al., 2013). Staphylococcal superantigens induce activation of polyclonal T cells and release of proinflammatory cytokines without antigenic peptide presentation. This effect occurs through their direct binding to the non-peptide groove of major histocompatibility complex class II (MHC II) molecules on dendritic cells and the T-cell receptor β chain on T cells (Arad et al., 2011). Arad et al. (2011) have expanded our understanding of superantigen-induced inflammation using murine models; however, clinical validation remains necessary to confirm these mechanisms in AD patients. Importantly, SEB can promote IL-31 secretion by Th2 cells, inhibiting keratinocyte differentiation and reducing filaggrin (FLG) expression, thereby impairing the skin barrier and intensifying pruritus (Sonkoly et al., 2006; Cornelissen et al., 2012). Superantigens can also act as allergens, triggering IgE-mediated responses that lead to histamine release from mast cells and basophils (Spaulding et al., 2013; Geoghegan et al., 2018).

Phenol-soluble regulatory proteins (PSMs), including α-type PSMs and δ-toxin, contribute significantly to the virulence of invasive *S. aureus* strains (Syed et al., 2015). Alpha-type PSMs trigger keratinocytes to release proinflammatory cytokines, exacerbating inflammation and skin damage characteristic of AD, as demonstrated in murine colonization models (Syed et al., 2015). In contrast, δ-toxin does not induce cytolysis but potently triggers mast cell degranulation, enhances the production of IgE and IL-4, and promotes allergic skin inflammation in a δ-toxin dependent manner (Nakamura et al., 2013; Kim et al., 2019a; Ogonowska et al., 2020). *S. aureus* α-toxin, or α-hemolysin, is a potent pore-forming cytolysin responsible for direct keratinocyte cytotoxicity, a process sexacerbated by Th2 cytokines (Berube and Bubeck Wardenburg, 2013; Brauweiler et al., 2014). Exposure to Th2 cytokines or keratinocytes derived from AD lesions enhances susceptibility to α-toxin-mediated cytotoxicity, a process dependent on the STAT6 signaling pathway (Brauweiler et al., 2014). Th2 cytokines suppress FLG and acid sphingomyelinase expression, leading to sphingomyelin accumulation on the keratinocyte surface, which acts as an α-toxin receptor (Brauweiler et al., 2014). This biochemical alteration significantly enhances the cytotoxicity of α-toxin against keratinocytes, thereby exacerbating epidermal damage and impairing skin barrier function (Brauweiler et al., 2014).

Protein A(SpA)and lipoproteins from *S. aureus* further aggravate the pathogenesis of AD by driving a key integrated signaling pathway — the TLR–TSLP–Th2 axis (comprising the Toll-like receptor, thymic stromal lymphopoietin, and T helper type 2 signaling cascade). These bacterial components activate keratinocyte tumor necrosis factor receptor-1 (TNFR-1) and Toll-like receptor 2 (TLR-2), triggering proinflammatory cytokine release, particularly TSLP and IL-8 (Vu et al., 2010; Classen et al., 2011). Additionally, TLR2-TLR6 heterodimer ligands in the membrane components of *S. aureus*, such as diacylated lipoproteins, can induce the secretion of thymic stromal lymphopoietin (TSLP) by keratinocytes (Vu et al., 2010). Functioning as a master regulator of Th2 immunity, TSLP orchestrates a multi-cellular inflammatory cascade: (1) On dendritic cells (DCs): TSLP-activated DCs, via OX40 ligand, drive the differentiation of naïve T cells into inflammatory TH2 cells that secrete IL-4, IL-5, IL-13, and TNF-α. These DCs selectively produce TH2-recruiting chemokines without generating IL-12, and further promote the expansion and polarization of allergen-specific TH2 memory cells (Ebner et al., 2007; Vu et al., 2010). (2) On other immune cells: TSLP directly acts on human mast cells, synergizing with IL-1 and TNF-α to promote the production of IL-5 and IL-13 (Allakhverdi et al., 2007). In human CD4^+^ T cells activated via T-cell receptor stimulation, TSLP significantly enhances their proliferative capacity (Rochman et al., 2007). For human eosinophils, TSLP prolongs survival and promotes the secretion of pro-inflammatory molecules (Wong et al., 2010). Furthermore, when human invariant natural killer T (iNKT) cells are co-cultured with dendritic cells, TSLP induces the expression of IL-4 and IL-13, along with further stimulation of IFN-γ production (Ck et al., 2010; Vu et al., 2010). Consequently, elevated TSLP levels sensitize individuals to environmental and *S. aureus*–derived allergens, amplifying AD symptoms (Ahmad-Nejad et al., 2004). More importantly, persistent colonization of *S. aureus* can further induce TSLP expression in keratinocytes, amplifying Th2-type inflammation and particularly promoting the differentiation of TH2/TNF double-positive inflammatory Th2 cells (Liu, 2006). Within the progressively intensified Th2-biased microenvironment, this TLR-driven TSLP–Th2 circuit establishes a self-perpetuating vicious cycle, which continuously fuels the progression and exacerbation of AD (Ebner et al., 2007).

Inflammasome activation also contributes to the inflammatory environment characteristic of AD. Studies have shown that *S. aureus* can activate the NLRP3 inflammasome in macrophages and epithelial cells, promoting proinflammatory cytokine secretion (Liu et al., 2021). In addition, *S. aureus* can activate the NLRP1 inflammasome in the skin, elevating IL-18 and IL-1β levels, particularly in genetically susceptible individuals carrying variants in IL1RL1, IL18R1, and IL18RAP, thus exacerbating AD severity (Vaher et al., 2023).

### 3.2 Cutaneous *Malassezia*

*Malassezia* species are lipophilic yeasts that are one of the most common fungi associated with AD, particularly on the head and neck, where skin lipids are abundant (Findley et al., 2013; Jo et al., 2016). By far the most common and abundant species are M. restricta and M. globosa (Gaitanis et al., 2012; Ianiri et al., 2022). However, several studies have found no significant difference in the number of *Malassezia* species present on the skin of healthy individuals and those with AD. This suggests that these fungi may be opportunistic organisms in AD (Sandström Falk et al., 2005; Yim et al., 2010; Kaga et al., 2011). AD patients have increased levels of *Malassezia*-specific IgE, and there is a correlation between AD severity and the amount of *Malassezia* species present. The level of *Malassezia* colonization increases up to 2–5 times in severe AD compared to mild or moderate cases (Kaga et al., 2011; Glatz et al., 2015; Nowicka and Nawrot, 2019). M. restricta, M. globosa, and M. sympodialis are the most commonly identified species in AD skin (Prohic et al., 2016; Lee et al., 2023).

Due to the compromised skin barrier commonly observed in AD patients, *Malassezia* species readily penetrate the epidermis, promoting colonization and infection. Once established, these fungi directly or indirectly interact with human keratinocytes and immune cells, initiating a robust inflammatory response (Nowicka and Nawrot, 2019; Thammahong et al., 2020).

The inflammatory response to *Malassezia* species primarily involves the activation of innate immunity through interactions with cell wall components. Components of the fungal cell wall bind to various receptors on host cell membranes, leading to ligand internalization and subsequent activation of multiple inflammatory pathways, including the NLRP3 inflammasome and downstream signaling cascades such as mitogen-activated protein kinase (MAPK), nuclear factor kappa B (NF-κB), and nuclear factor of activated T cells (NFAT) (Sparber and LeibundGut-Landmann, 2017; Park HR. et al., 2021; Lee et al., 2023). Moreover, *Malassezia* are recognized by Toll-like receptors (TLR) on human keratinocytes and dendritic cells and activate inflammation by inducing the secretion of anti-microbial peptides and cytokines (e.g., human bdefensin2 and CXCL8) (Baroni et al., 2006; Brasch et al., 2014). Additionally, *Malassezia* species activate human dendritic cells via the NLRP3 inflammasome, leading to increased production of IL-1b, IL-4, IL-5, and IL-13 (Kistowska et al., 2014).

Beyond cell wall components, secreted factors and metabolic products from *Malassezia* further exacerbate skin inflammation. These fungi produce proteases, toxic metabolites, and reactive oxygen species (ROS), promoting inflammatory signaling in keratinocytes through NF-κB activation. Research has shown that *M. sympodialis*-derived extracellular nanovesicles can activate skin dendritic cells and dermal mast cells, releasing TNF-a, IL-6, IL-8, IL-10 and IL-12p70, which can aggravate inflammatory responses (Gehrmann et al., 2011).

Among these inflammatory pathways, the IL-23/Th17 axis has emerged as a critical mediator in *Malassezia*-induced cutaneous inflammation. Accumulating evidence indicates that IL-23 and IL-17 pathways play pivotal roles in amplifying *Malassezia*-induced skin inflammation in murine models, with IL-22 also being markedly upregulated in response to *Malassezia* exposure (Sparber et al., 2019). Further *in vivo* studies demonstrated that *Malassezia globosa* robustly stimulates IL-23 production by keratinocytes via activation of the TLR2/MyD88/NF-κB signaling cascade (Jia et al., 2024). IL-23, a cytokine crucial for pathogenic Th17 cell differentiation, significantly increases the proportion of pathogenic Th17 subsets, exacerbating inflammation. Conversely, blocking IL-23 signaling markedly reduces pathogenic Th17 differentiation, highlighting the IL-23/Th17 axis as a key immunological mechanism underlying *Malassezia*-driven skin inflammation (Jia et al., 2024).

### 3.3 Skin microbiota-host interactions in AD

Most microbes residing on the skin behave as commensal or mutualistic under steady-state conditions, playing a crucial role in maintaining skin homeostasis (Dethlefsen et al., 2007). Among the dominant skin colonizers are coagulase-negative Staphylococcus species (CoNS), including *Staphylococcus epidermidis* (*S. epidermidis*), *Staphylococcus hominis*, and *Staphylococcus lugdunensis*, with *S. epidermidis* being the most prominent. *S. epidermidis* primarily functions as a mutualist but can act as an opportunistic pathogen in certain contexts (Hon et al., 2016). Notably, the severity of AD is inversely correlated with the abundance of *S. epidermidis* relative to *S. aureus (*Byrd et al., 2017). The interactions between *S. epidermidis* and *S. aureus* exhibit pronounced strain-dependent and context-dependent characteristics. On one hand, certain *S. epidermidis* strains can inhibit *S. aureus* biofilm formation and colonization by secreting AMPs, PSMs, as well as autoinducing peptides that interfere with *S. aureus* quorum sensing (Cogen et al., 2010; Gonzalez et al., 2021). Studies have also shown that certain *S. epidermidis* strains can secrete the serine protease glutamyl endopeptidase (Esp), thereby inhibiting *S. aureus* biofilm formation and colonization. But this effect is largely strain- and context-dependent and has been mainly observed *in vitro*, requiring further *in vivo* validation (Iwase et al., 2010). These antagonistic activities may help limit pathogen overgrowth, maintain skin microbial homeostasis, and activate host immune responses, thereby further inhibiting *S. aureus* growth. Conversely, when paired strains of *S. epidermidis* and *S. aureus* from the same individual were co-cultured, 13% of the subjects exhibited cooperative formation of mixed-species biofilms, in which the total biofilm biomass even exceeded the sum of the biofilms formed by each species individually. These findings indicate that *S. epidermidis* can inhibit, coexist with, or even enhance *S. aureus* biofilm formation (Gonzalez et al., 2021). This suggests that under certain conditions, *S. epidermidis* could facilitate pathogen persistence and skin inflammation. Moreover, the growth mode of *S. epidermidis* (biofilm vs planktonic) appears to differentially influence skin immune responses. Naik et al. demonstrated in a mouse model that *S. epidermidis* can induce commensal-specific T cells to home to the epidermis and promote keratinocyte secretion of calprotectin (S100A8/S100A9) via an IL-17A–dependent pathway (Naik et al., 2015). However, Gonzalez et al. observed that the biofilm propensity of *S. epidermidis* was negatively correlated with S100A8/S100A9 expression in both non-lesional and lesional skin, suggesting that biofilm-forming *S. epidermidis* is less efficiently taken up by skin-resident dendritic cells for presentation to commensal-specific T cells (Gonzalez et al., 2021). These findings highlight the dual role of *S. epidermidis*: while it may confer protective effects on skin health, it can also act as a “context-dependent opportunistic promoter” depending on strain-specific traits and the local microenvironment, underscoring the need for future studies to address strain-level variability and the regulatory influence of complex *in vivo* conditions.

The interactions between fungi and bacteria also play a significant role in the pathogenesis of AD. *Malassezia* species, common lipophilic commensal fungi of the skin, have been reported to increase in abundance in AD lesions, particularly on the head and neck, and may contribute to disease by activating Th17/Th22-associated inflammatory pathways (Gori et al., 2023; Chong et al., 2024). In contrast, *S. aureus* is primarily associated with Th2-driven immune responses (Berube and Bubeck Wardenburg, 2013). Although studies investigating the potential synergistic or antagonistic relationship between *Malassezia* and *S. aureus* remain limited, existing evidence suggests that these microbes may influence each other through competition for colonization sites on the skin or by modulating the local immune environment. Notably, *Malassezia* and *S. aureus* share ecological niches at multiple sebaceous skin sites as well as in the anterior nares, and *S. aureus* colonization in the nasal cavity typically depends on biofilm formation (Iwase et al., 2010; Oh et al., 2014). In fact, *M. globosa* is widely distributed on healthy skin and constitutes an important component of the stable skin microbiota in adults (Oh et al., 2014). Studies have shown that the aspartyl protease MgSAP1 secreted by *M. globosa* exhibits high cleavage activity at peptide bonds between lysine and aromatic residues, and the key virulence protein of *S. aureus*, SpA, is rich in lysine, making it an ideal substrate for MgSAP1. SpA is a key mediator of *S. aureus* biofilm formation and can also inhibit phagocytosis by host immune cells. MgSAP1 degrades SpA, directly disrupting the ‘scaffold’ or adhesion factors essential for biofilm formation, thereby exhibiting significant antagonistic potential. *In vitro* experiments further demonstrated that MgSAP1 rapidly degrades recombinant SpA and markedly reduces the *in situ* biofilm volume of *S. aureus* without affecting bacterial viability (Ianiri et al., 2018; Li et al., 2018). These findings reveal a potential mechanism by which *M. globosa* regulates interactions between commensal and opportunistic pathogenic bacteria through protease secretion, and suggest that fungal–bacterial interkingdom crosstalk may play an important role in the microbial ecology and immune regulation of AD.

Integrated multi-omics analyses have revealed critical insights into microbial dysbiosis and its relevance to host responses in AD. A large cohort (n = 316) combining analysis of skin microbiomes and associated transcriptomes found a notable loss of strictly anaerobic bacteria in AD, suggesting a switch in the skin microbiome from anaerobic to aerobic metabolism (Fyhrquist et al., 2019). By integrating microbial composition with global gene expression patterns, the study demonstrated that AD skin is dominated by *Staphylococcus aureus* and associated with distinct transcriptomic signatures enriched for skin barrier dysfunction, tryptophan metabolism, and immune activation. In contrast, psoriasis exhibited more diverse microbial communities with weaker associations to host gene expression. These findings highlight the power of multi-omics approaches to elucidate host–microbe interactions and identify disease-specific biomarkers in inflammatory skin disorders. Gram-positive anaerobic cocci (GPAC) such as *Finegoldia*, *Anaerococcus*, and *Peptoniphilus* can rapidly induce AMP responses in human keratinocytes. This may serve as an important signaling mechanism when symbiotic bacteria come into close contact with epidermal keratinocytes after skin injury. Partial or complete loss of *Finegoldia magna* could impair or delay this danger signaling, potentially facilitating *S. aureus* colonization or infection (Zeeuwen et al., 2017).

These insights underscore the value of multi-omics approaches in understanding the skin microbiome–host interface. Beyond microbial composition, microbial metabolites such as short-chain fatty acids (SCFAs) and indole derivatives—particularly those produced by commensal anaerobes—are emerging as critical regulators of skin homeostasis (Yang et al., 2025). These metabolites can modulate immune responses and barrier integrity through host receptors such as the aryl hydrocarbon receptor (AHR), influencing keratinocyte differentiation, AMP expression, and cytokine production (Liu et al., 2020; Accioli C de et al., 2023). Altered metabolite profiles in AD skin may thus exacerbate inflammation or compromise barrier repair. Integrating metagenomic, metabolomic, and transcriptomic data will be essential for dissecting these complex host–microbial–metabolite interactions and identifying novel therapeutic targets.

These alterations in commensal populations, including the loss of anaerobic bacteria and the overgrowth of pathogens like *S. aureus*, trigger complex host responses. While the pathogenic mechanisms of *S. aureus*—including toxin production and inflammasome activation—have been well-characterized, the host response remains equally critical in modulating disease severity (Kobayashi et al., 2015; Fyhrquist et al., 2019). In response to *S. aureus*, the host upregulates expression of β-defensins (e.g., DEFB4) and members of the S100 protein family, including S100A8, S100A9, and S100A7. β-defensin acts as a potent AMP and chemoattractant for leukocytes, activating antigen-presenting cells to enhance local immune responses and modulate adaptive immunity (Röhrl et al., 2010). Psoriasin (S100A7), another keratinocyte-derived AMP, preferentially kills *E. coli* and is markedly increased (up to 1500-fold) on the skin surface of AD patients compared to healthy skin (Gläser et al., 2009). This elevation is consistent with tape stripping experiments in healthy individuals, suggesting that skin barrier disruption is a key driver for increased psoriasin expression in AD. Calprotectin, a heterodimer formed by S100A8 and S100A9, plays a complex role in the microbial-host interplay. While calprotectin inhibits microbial colonization by sequestering zinc and manganese, biofilm formation by *S. epidermidis* may suppress this antibacterial mechanism, allowing mixed biofilms of *S. aureus* and *S. epidermidis* to establish (Gonzalez et al., 2021).

A genomic and molecular comparison of non-lesional, acute, and chronic AD lesions revealed significant upregulation of S100A7, S100A8, and S100A9 in both acute and chronic phases (Gittler et al., 2012). Notably, the expression of these S100 proteins, particularly in lesional skin, has been shown to correlate positively with clinical disease severity scores such as SCORAD (Szegedi et al., 2015; Dessie et al., 2024). These S100 proteins are critically involved in inflammation through chemotaxis of T cells, monocytes, and neutrophils, and exhibit pro-inflammatory functions in various inflammatory diseases (Roth et al., 2003; Eckert et al., 2004; Goyette and Geczy, 2011). Although possessing antimicrobial activity their elevated expression in AD does not prevent the high prevalence of *S. aureus* infections, implying that their pro-inflammatory effects predominate (Gittler et al., 2012), and that they may serve as both biomarkers of inflammation and drivers of the chronic inflammatory loop. The upregulation of S100 proteins closely associates with inflammatory cytokines IL-22 and IL-17, mainly secreted by Th22 and Th17 cells (Boniface et al., 2005; Nograles et al., 2008; Gittler et al., 2012). IL-22 and IL-17 synergistically enhance S100 protein expression in keratinocytes. The increased levels of S100A8/A9 further promote recruitment of inflammatory cells, amplifying local immune responses and establishing a positive feedback loop that perpetuates inflammation (Gittler et al., 2012).

Beyond disturbances in the cutaneous microbiota, impairments in intestinal barrier function and dysregulation of gut microbial communities may substantially influence global skin homeostasis (Salem et al., 2018; Chen et al., 2024). The gut–skin axis encompasses a highly coordinated, bidirectional communication system linking intestinal and cutaneous tissues (De Pessemier et al., 2021). The gut microbiota primarily modulates systemic and local inflammation through interactions with the immune system, thereby influencing skin health (Polkowska-Pruszyńska et al., 2020). The gut microbiota primarily maintains intestinal barrier integrity by converting indigestible complex polysaccharides into vitamins, particularly vitamin K and B12, as well as SCFAs, notably butyrate and propionate (LeBlanc et al., 2013). SCFAs are key immunoregulatory molecules that promote colonic immune balance by activating and expanding regulatory T cells (Tregs) and suppressing proinflammatory responses. They also limit inflammation by reducing immune cell migration, reactive oxygen species, cytokine release, and NF-κB/TNF-α signaling (Hu et al., 2021; Kanda et al., 2021). Furthermore, in patients with AD, supplementation with *Bifidobacteria* has been shown to increase fecal tryptophan metabolite levels, alleviate pruritus, improve quality of life, and enhance colonic mucosal function (Tian et al., 2021). Different probiotic strains can elicit diverse immune responses: some strains activate the immune system by promoting the production of IL-12, IL-18, and TNF-α, while others enhance the secretion of IL-10 and TGF-β, thereby exerting anti-inflammatory effects and offering potential strategies for managing AD (Cukrowska et al., 2010). This immune milieu also facilitates the induction of inducible Tregs, which play a critical role in maintaining peripheral immune tolerance by balancing effector T cells and Tregs (Sheikhi et al., 2017). Interestingly, studies in mouse models have indicated that the skin microbiome may influence the severity of inflammatory bowel disease (IBD). In particular, the common commensal skin fungus *Malassezia restricta* has been associated with Crohn’s disease, with increased abundance correlating with greater disease severity and exacerbation of colitis (Limon et al., 2019).

**Figure 2:**
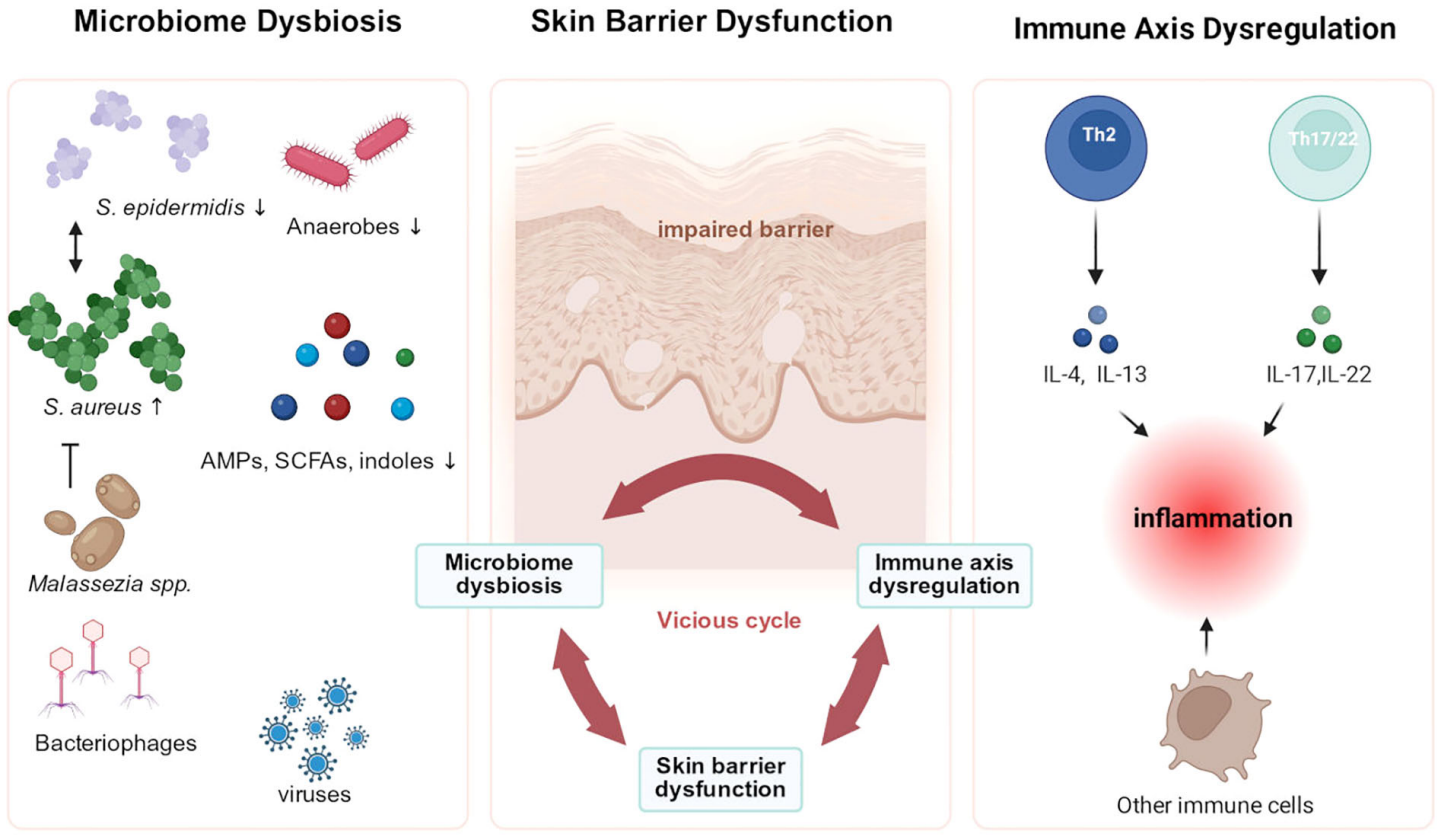
Interplay among microbiome dysbiosis, skin barrier dysfunction, and immune axis dysregulation in atopic dermatitis (AD). Barrier disruption facilitates microbial imbalance, characterized by a reduction in commensal microorganisms, overgrowth of *Staphylococcus aureus* (*S. aureus*), alterations in fungal and viral community structures, and decreased levels of certain microbe-derived metabolites, including antimicrobial peptides (AMPs), short-chain fatty acids (SCFAs), and indole derivatives. In turn, microbial dysbiosis further compromises barrier integrity and aberrantly activates immune responses. Immune dysregulation, dominated by Th2 and Th17/Th22 polarization, with contributions from other immune cells, amplifies inflammatory signaling and suppresses epidermal differentiation and lipid synthesis, thereby exacerbating barrier damage. Together, these processes form a self-perpetuating vicious cycle that drives the persistence and progression of chronic inflammation in AD. *S. aureus*, Staphylococcus aureus; *S. epidermidis, Staphylococcus epidermidis*; AMPs, antimicrobial peptides; SCFAs, short-chain fatty acids.

This complex interplay between protective microbial factors and pathogenic mechanisms highlights how shifts in the skin microbiome, along with host immune dysregulation, contribute to the development and exacerbation of AD (**Figure 2**).

### 3.4 Virome and phageome in AD

Beyond bacteria and fungi, recent research has begun to uncover the significance of the skin virome—particularly bacteriophages (phages)—in regulating microbial ecology and influencing skin health (Dominguez-Lafage et al., 2023). The virome, composed of eukaryotic viruses and phages, represents an underexplored yet dynamic component of the skin microbiome. In AD, disruptions in the bacterial community are well documented; however, emerging evidence suggests that the virome may also play a key modulatory role (Fuxench et al., 2023).

Phages, the viruses that infect bacteria, can shape bacterial populations through lytic activity or lysogenic conversion. In AD, *Staphylococcus aureus* is a dominant pathogen whose proliferation may be influenced by its associated phages (Wang et al., 2024). Lytic phages targeting *S. aureus* could contribute to bacterial population control and influence the outcomes of host–microbe interactions (Plumet et al., 2022). Conversely, lysogenic phages integrated into bacterial genomes may carry virulence factors, such as toxin genes, and promote horizontal gene transfer, thereby enhancing bacterial fitness and pathogenic potential (Baaziz et al., 2024). This dual role highlights the complexity of phage–bacteria–host interactions in AD.

Moreover, shifts in phage populations may act as a marker or driver of microbial dysbiosis. Some studies have shown that viral richness and diversity are reduced in AD skin, potentially reflecting a collapse of phage-mediated bacterial regulation (Bjerre et al., 2021). Others have proposed that phage dynamics may respond to environmental factors, inflammation, or therapeutic interventions such as antibiotics or emollients, thereby modulating the skin’s microbial equilibrium (Ghadimi et al., 2024).

Eukaryotic viruses, although less well studied, may also contribute to the inflammatory environment in AD. For example, increased prevalence of human papillomavirus (HPV), polyomaviruses, and herpesviruses has been observed in AD skin, possibly due to barrier disruption and local immune suppression (Morgan et al., 2015; Novak and Cabanillas, 2022). These viruses may interact with host immunity and further amplify disease progression, although their exact role remains to be elucidated.

Taken together, the skin virome and phageome represent emerging layers of complexity in AD pathogenesis. Integrating virome profiling into multi-omics frameworks could uncover novel biomarkers, inform therapeutic strategies—such as phage therapy—and enhance our understanding of microbial–host co-regulation in chronic skin inflammation.

## 4 Therapeutic approaches on skin microbiome composition

The pathogenesis of AD is multifactorial, involving a complex interplay between genetic predisposition, environmental factors, and immunological responses. In recent years, advances in microbiome research have unveiled significant insights into the role of skin microbiome in modulating inflammatory pathways and maintaining barrier function. Emerging evidence suggests that dysbiosis, or microbial imbalance, may contribute to the exacerbation of AD symptoms, highlighting microbiome-targeted interventions as promising therapeutic strategies.

In a prospective pilot study, researchers conducted skin microbiota transplantation in four healthy volunteers by collecting microbiota from the forearm and transferring it unidirectionally to the back using culture-based methods and 16S rRNA V1–V3 deep sequencing. Comparison of baseline and post-transplant communities revealed that DNA from some bacterial species persisted temporarily at the recipient site, indicating that transfer of bacteria from one skin site to another is technically feasible. However, due to uncertainty regarding the survival and colonization of live bacteria within the entire microbial community, as well as inter-individual variability in skin microbiota, whole-community transplantation still faces challenges for clinical application. Furthermore, the safety of allogeneic skin microbiome transplantation remains a major concern, particularly on barrier-compromised AD skin, with potential risks including the transfer of opportunistic pathogens or unintended immune activation (Jw et al., 2019). Based on these limitations, recent research has increasingly focused on the targeted application of functional commensal strains—specifically, skin commensals with antimicrobial activity against pathogens such as *Staphylococcus aureus*—applied topically to the skin of AD patients. Compared with whole-community transplantation, this strategy allows more controlled modulation of the skin microbiome and targeted suppression of pathogenic colonization, highlighting its potential therapeutic value (Perin et al., 2019).

Interactions between nonpathogenic bacteria, such as the resident skin microbiota, and host cells, as well as the immune regulation they mediate, may offer novel therapeutic strategies for modulating cutaneous inflammatory responses. Gueniche et al. conducted a randomized, double-blind, placebo-controlled clinical trial in which a 5% *Vitreoscilla filiformis* (*V. filiformis*) lysate was topically applied to the skin of 75 patients with mild AD to evaluate its therapeutic effects. The study demonstrated that the 5% *V. filiformis* lysate significantly improved SCORAD scores, alleviated pruritus, and consequently improved sleep quality. As the study population comprised patients with mild AD, for whom sleep disturbances were not a major symptom, these findings suggest that more pronounced benefits may be observed in patients with more severe disease. Microbial colonization analyses indicated that *V. filiformis* lysate reduced *S. aureus* colonization on the skin, and efficacy was observed even in the absence of marked pathogenic bacterial colonization. The underlying mechanisms may involve induction of AMPs and other innate immune responses, as well as potential immunomodulatory or immunoregulatory effects (Gueniche et al., 2008). However, the study was limited by its small sample size, inclusion of only mild AD cases, short follow-up period, and lack of direct verification of immunological mechanisms, which constrains the generalizability of the findings. Future large-scale, long-term, mechanism-oriented studies are warranted to further validate its clinical utility.

Myles IA et al. demonstrated in mouse experiments that specific mucosal strains of *Roseomonas mucosa* (R. mucosa) isolated from healthy volunteers (HV) improved disease outcomes in AD mouse and cell culture models, whereas strains derived from AD patients worsened disease. Based on these findings, the research team conducted the first-in-human clinical trial, applying HV-derived R. mucosa topically to adults and children with mild-to-moderate AD. The results showed that R. mucosa treatment significantly improved local Eczema Area and Severity Index (EASI) scores, reduced *S. aureus* colonization, and decreased the need for topical corticosteroids. The potential mechanisms include enhancement of epithelial barrier function, modulation of innate and adaptive immune balance, and inhibition of *S. aureus* growth. Metabolomic analyses further suggest that lipid mediators and other biochemical markers may serve as potential targets for assessing host skin metabolic changes in future studies. No serious adverse events were observed, and the treatment was well tolerated (Myles et al., 2018). However, the study had a limited sample size, the microbiome and underlying mechanisms were not comprehensively validated, and the long-term efficacy as well as the impact of environmental factors on the microbial community remain unclear.

Similarly, Nakatsuji et al. conducted a randomized clinical trial in adults with AD positive for *S. aureus* (SA), demonstrating that topical application of autologous or healthy donor-derived coagulase-negative staphylococci with anti-SA activity (CoNS-AM+) significantly reduced SA colonization on lesional skin and improved local EASI scores. The treatment was well tolerated, with only mild adverse events reported. Limitations of the study include its single-center, small-sample design, restriction to SA-positive adult AD patients, and lack of direct mechanistic validation (Nakatsuji et al., 2021a).

To further validate these findings, Nakatsuji et al. performed a high-throughput functional screening of CoNS isolated from the skin of healthy individuals and patients with AD. They identified specific *Staphylococcus hominis* (*S. hominis*) strains capable of producing novel lantibiotics with potent and highly selective activity against *S. aureus*, which significantly reduced *S. aureus* colonization in ex vivo pigskin models, murine skin, and the skin of patients with AD. Further characterization revealed that these strains secrete two previously unrecognized antimicrobial peptides, Sh-lantibiotic-α and Sh-lantibiotic-β, which not only inhibit multiple *S. aureus* strains, including methicillin-resistant isolates, but also exhibit “precision antimicrobial” activity with minimal effects on other skin commensals. Moreover, these bacterially derived peptides act synergistically with the host antimicrobial peptide LL-37, suggesting that host and commensal microbes cooperatively contribute to the cutaneous defense system. Notably, these protective commensal strains and their antimicrobial products are markedly reduced in patients with AD, which may partly explain their increased susceptibility to *S. aureus* colonization. In subsequent clinical exploration, topical application of *S. hominis* A9 (ShA9) reduced *S. aureus* burden on lesional skin and improved EASI scores in a subset of patients, although the overall difference did not reach statistical significance. Nevertheless, these studies are limited by small sample sizes, short follow-up periods, and incomplete mechanistic characterization, particularly regarding the molecular targets of lantibiotics, the potential for resistance development, and their effects on host immune responses (Nakatsuji et al., 2021b).

Collectively, these findings not only elucidate how dysbiosis of the skin microbiome contributes to disease progression but also underscore the therapeutic potential of utilizing skin symbiotic bacteria for AD management.

Microbiome-targeted interventions, including probiotics, prebiotics, and postbiotics, have increasingly attracted attention in therapeutic research (Prajapati et al., 2025). Multiple meta-analyses have indicated that probiotic supplementation confers an overall benefit in children with AD (da Costa Baptista et al., 2013; Huang et al., 2017; Chang et al., 2017). For example, studies have shown that *Lactobacillus acidophilus* DDS-1 and *Bifidobacterium lactis* UABLA-12 can improve moderate-to-severe AD in children (Gerasimov et al., 2010), while *Lactobacillus salivarius* also provides short-term benefits in moderate-to-severe AD (Wu et al., 2012), with these effects potentially linked to restoration of the gut microbiota (Drago et al., 2011). In addition, oral administration of *Lactobacillus acidophilus* L-92–derived prebiotics has been shown to improve AD in Japanese children by modulating the Th1/Th2 immune axis (Torii et al., 2011), and synbiotics (combining probiotics and prebiotics) have also demonstrated potential as an adjunctive therapy (Hy et al., 2017). Postbiotics, as microbial metabolites or bacterial components, offer a higher safety profile compared with live biotherapeutics (Prajapati et al., 2025). Kim et al. demonstrated that MB-2006, a co-fermented product of *Smilax china* extract with *Lactobacillus acidophilus* and *Lactobacillus rhamnosus*, was more effective than the individual probiotics in suppressing IL-4, TSLP, and NF-κB pathway activation (Kim et al., 2024). It should be noted that current meta-analyses still exhibit heterogeneity and potential publication bias, and most clinical trials have small sample sizes with short follow-up periods, so the evidence for efficacy remains to be further validated.

**Figure 3:**
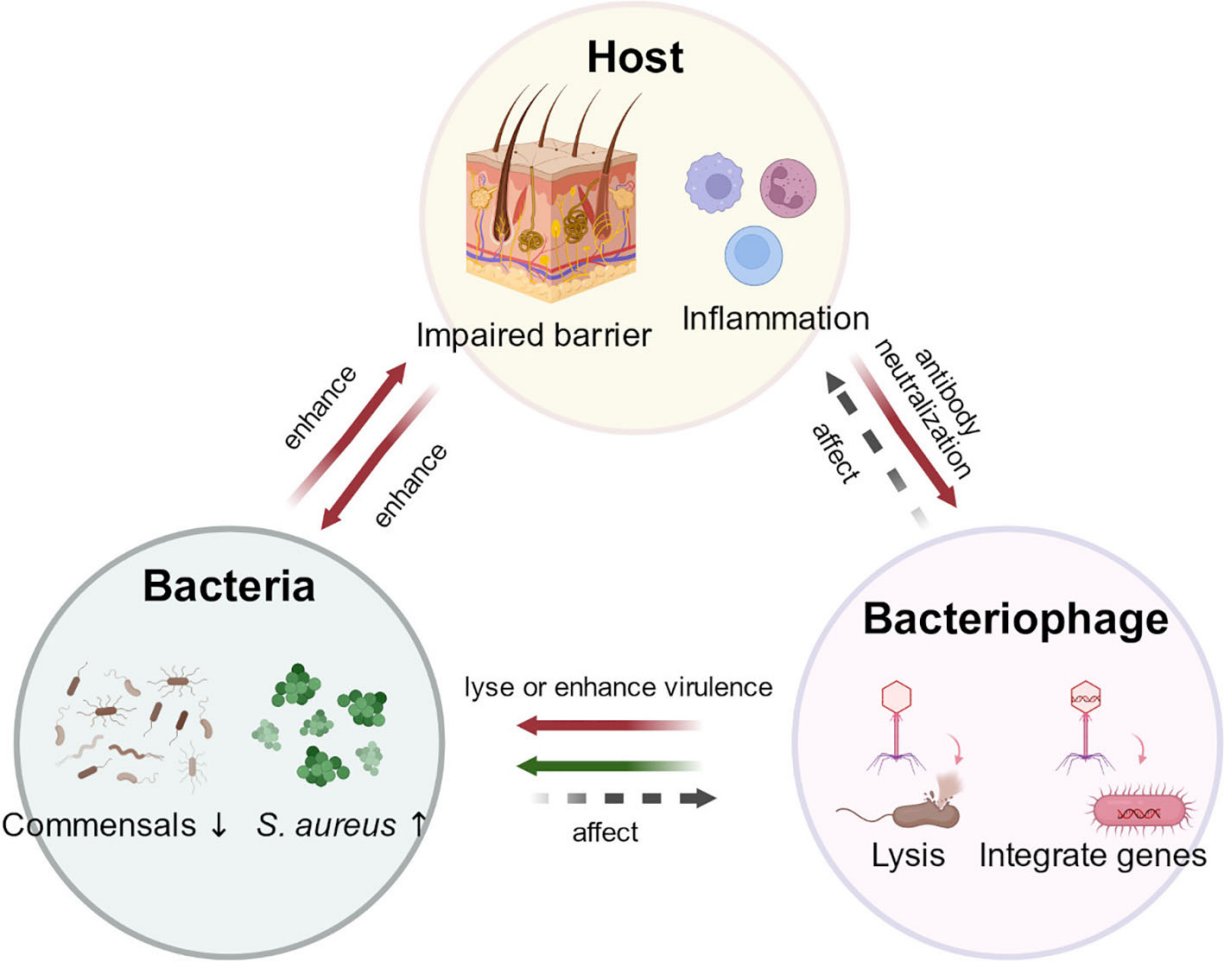
Dynamic interactions among bacteriophages, bacteria, and the host in atopic dermatitis (AD). Lytic phages can lyse *S. aureus*, reducing bacterial burden, whereas lysogenic phages may enhance bacterial virulence. *Staphylococcus aureus* (*S. aureus*) promotes inflammation and disrupts epidermal barrier function, while dysregulation of the host immune axis can lead to bacterial community imbalance. Meanwhile, host-derived neutralizing antibodies may limit phage activity, and changes in the phage community may respond to host inflammation, thereby modulating bacterial community homeostasis in the skin. *S. aureus*, *Staphylococcus aureus*.

*S. aureus* colonization is a key feature of the skin in patients with AD. During acute infection, antibiotics are commonly used; however, most *S. aureus* strains exhibit multidrug resistance, and the non-specific action of antibiotics may further exacerbate dysbiosis of the skin microbiome (Castillo-González et al., 2022). *Bacteriophages*, natural viruses that specifically target bacteria, have been used for over a century to treat various acute and chronic bacterial infections. Beyond their direct potential to suppress pathogenic bacteria, emerging evidence suggests that endogenous bacteriophage communities (the phageome) and their dysbiosis may influence skin health. Phage dysbiosis has been observed in lesional skin of patients with psoriasis and acne (Barnard et al., 2016; Wang et al., 2020). Emerging research also suggests that phage dysbiosis may contribute to microbial imbalances in dermatologic diseases such as psoriasis and acne (Natarelli et al., 2023), raising the possibility that phage dysbiosis could also be relevant in AD, although this hypothesis requires further investigation. Therefore, topical bacteriophage therapy represents a promising and innovative direction for AD treatment, particularly in addressing *S. aureus* colonization (**Figure 3**).

Yuzuki et al. isolated a novel bacteriophage, SaGU1, from sewage samples in Gifu, Japan, in an *in vitro* study. SaGU1 specifically lysed *S. aureus* derived from the skin of AD patients without affecting the commensal bacterium *S. epidermidis*. Genomic analysis revealed that SaGU1 harbors neither virulence factors nor antibiotic resistance genes, and stability testing demonstrated good thermal stability under both physiological and acidic conditions (Shimamori et al., 2021). These findings suggest that SaGU1 represents a promising candidate for the development of phage therapy targeting pathogenic *S. aureus* in the context of AD (Shimamori et al., 2020; Shimamori et al., 2021).

Researchers have utilized a bacteriophage product prepared according to good manufacturing practices (GMP) as an adjunctive therapy to antibiotics in 13 patients with severe *S. aureus* infections. During or after treatment, bacterial load and inflammatory response were reduced, and no phage-resistant strains of *S. aureus* were isolated. No adverse events were reported during the process (Górski et al., 2017; Petrovic Fabijan et al., 2020). The topical application of phage may prove an efficacious treatment for chronic non-healing wounds associated with *Escherichia coli*, *S. aureus* and *Pseudomonas aeruginosa* (Gupta et al., 2019). Kim et al. evaluated the effects of topical application of the staphylococcal bacteriophage pSa-3 in an *S. aureus*–infected AD mouse model. The results showed that a single-day treatment with pSa-3 (10^7^ PFU) alone did not significantly reduce the bacterial load on the skin, whereas consecutive three- or five-day phage treatments significantly decreased colony-forming units (CFUs). Moreover, the combined application of pSa-3 with Tween 20 further enhanced the antibacterial effect and downregulated the expression of pro-inflammatory cytokine genes, including IL-1β, IL-12, and IFN-γ (Kim et al., 2020). Despite its promise, phage therapy, as a highly specific microbiome-targeted antimicrobial strategy, faces several unique translational challenges. Regulatory pathways for live, evolving biological agents are not well-established, creating significant hurdles for clinical approval compared to conventional drugs (Furfaro et al., 2018; Yang et al., 2023). Furthermore, the host immune system can produce neutralizing antibodies against phages, potentially limiting the efficacy of repeated treatments. The development of bacterial resistance to phages, though manageable with phage cocktails, also remains a concern (Hyman and Abedon, 2010). Crucially, there is a significant gap in clinical evidence, with a scarcity of large-scale, randomized controlled trials to definitively establish safety and efficacy in AD patients (Luong et al., 2020; Uyttebroek et al., 2022). Addressing these regulatory, immunological, and clinical evidence gaps is essential for phage therapy to move from compassionate use to mainstream clinical practice.

Although interventions targeting the skin microbiome show promising potential in the treatment of AD (**Table 3**), their clinical application still faces several common challenges. First, the skin microbiome of AD patients exhibits a high degree of individual variability, resulting in significant differences in responses to the same microbial intervention among different individuals. Achieving precise and personalized interventions remains an urgent issue to be addressed. Second, safety concerns cannot be overlooked, especially in cases of long-term use or in individuals with compromised immune function, where microbial interventions may trigger unintended immune reactions or dysbiosis. Currently, microbial formulations lack standardized protocols in strain selection, formulation stability, storage and transportation conditions, and usage frequency, highlighting the need for establishing standardized production and evaluation systems. Moreover, the cost-effectiveness of such therapies remains unclear, particularly when compared to traditional treatment modalities, necessitating systematic economic evaluations.

**Table 3:** Summary of key clinical trials on skin microbiome–targeted interventions for atopic dermatitis.

Study	Design	Intervention	Control	Sample size	Primary outcomes	Key results	Limitations
Gueniche et al (Gueniche et al., 2008)	RCT	V. filiformis cream 5%	vehicle cream	75	SCORAD score; TEWL; itch; sleep; assessment of microflora.	significantly decreased SCORAD and pruritus; decreased colonization of *S. aureus;*	only mild AD included, short follow-up, mechanisms unverified, microbiology limited to S. aureus without community-wide sequencing.
Myles et al (Myles et al., 2018)	open-label, single-arm study	Roseomonas	NS	15	SCORAD score; adverse events; CDLQI;	No adverse events; significantly decreased SCORAD score	Limited sample size; microbiome and mechanisms not fully validated; long-term efficacy and environmental impacts on microbiota remain unclear.
Nakatsuji et al (Nakatsuji et al., 2021a)	RCT	CoNS-AM^+^	vehicle	11	*S. aureus* abundance; EASI; adverse events;	no serious adverse events; decreased colonization of *S. aureus;* decreased EASI score	Single-center, small sample, limited to *S. aureus*-positive adult AD, mechanism not directly verified.
Nakatsuji et al (Nakatsuji et al., 2021b)	RCT	Staphylococcus hominis A9	vehicle	54	adverse event rate; colony-forming units of *S. aureus*;	well-tolerated; decreased colonization of *S. aureus;*	Small sample size, short follow-up, incomplete elucidation of lantibiotic mechanism

At present, research in this field is still predominantly based on animal studies, with limited clinical trials available. These trials generally suffer from small sample sizes, short follow-up durations, and a lack of long-term safety and tolerability data. Therefore, future efforts should focus on conducting large-scale, multicenter, long-term clinical trials to comprehensively assess efficacy and safety. Concurrently, accelerating the development of robust quality control and regulatory frameworks is essential to facilitate the clinical translation and standardized application of microbiome-based interventions.

## 5 Conclusion

Atopic dermatitis pathogenesis is best understood not as a linear cascade but as a self-perpetuating vicious cycle involving skin barrier dysfunction, immune dysregulation, and microbial dysbiosis. At the heart of this pathological network lies *Staphylococcus aureus*, whose dominance reinforces both inflammation and barrier impairment. This recognition has shifted therapeutic priorities—from broad immunosuppression toward targeted ecological restoration—where restoring microbial homeostasis through microbiome-based interventions offers a promising new paradigm. Strategies such as live biotherapeutic products and precision phage therapy exemplify this shift, offering innovative avenues for sustainable disease control.

However, realizing the full potential of these therapies requires a clearly defined research roadmap. First, efforts must move beyond species-level associations to uncover strain-specific mechanisms through integrated multi-omics approaches (Nakajima et al., 2024). Second, understanding how specific microbial lineages and their metabolic outputs modulate AD endotypes is crucial for patient stratification. For instance, future clinical practice could involve using metabolomic or proteomic profiles—such as specific lipid signatures or tryptophan metabolite levels—to classify patients into distinct inflammatory endotypes (e.g., Th2-high vs. Th17/22-dominant) (Fukushima-Nomura et al., 2025). This stratification would, in turn, guide the selection of personalized interventions, such as prescribing AHR agonists for patients with deficient indole production or selecting specific bacteriotherapies to counteract pathogenic metabolic activities. Third, there is a need to develop next-generation biotherapeutics, including engineered probiotics with enhanced immunomodulatory functions and multi-kingdom approaches that incorporate the roles of the cutaneous mycobiome and phageome in skin ecosystem regulation.

In conclusion, microbiome-targeted strategies hold great promise for transforming the prevention and treatment of atopic dermatitis. Success will hinge on collaborative, multidisciplinary efforts spanning dermatology, microbiology, immunology, and bioinformatics. By deepening our understanding of microbe–host interactions and translating these insights into precision-guided ecological interventions, we can move closer to not only managing symptoms, but truly restoring long-term skin health—and breaking the vicious cycle of AD for good.

Key translational messages from this review include:

- A paradigm shift from broad microbial suppression (e.g., antibiotics) toward targeted ecological restoration is essential for long-term disease management.
- Emerging targeted microbiome therapies aim to reconstruct functionally intact microbial communities rather than merely suppress microorganisms.
- Effective microbiome-based therapies require strain-level precision, as functional properties (protective vs. pathogenic) vary significantly even within the same species.
- Integrating multi-omics data (metagenomics, metabolomics, transcriptomics) is critical for stratifying patients and developing personalized interventions based on functional biomarkers.
- Overcoming regulatory, manufacturing, and safety challenges is paramount for the successful clinical translation of live biotherapeutic products and phage therapies.
